# Comparison of hierarchical EMAX and NDLM models in dose-response for early phase clinical trials

**DOI:** 10.1186/s12874-020-01071-2

**Published:** 2020-07-20

**Authors:** Xiaqing Huang, Byron J. Gajewski

**Affiliations:** Department of Biostatistics & Data Science, University of Kansas Medical Center, Mail Stop 1026, 3901 Rainbow Blvd., Kansas City, KS 66160 USA

**Keywords:** Dosing design, Bayesian models, Predicting phase III, Logistic models, Flexible models

## Abstract

**Background:**

Phase II clinical trials primarily aim to find the optimal dose and investigate the relationship between dose and efficacy relative to standard of care (control). Therefore, before moving forward to a phase III confirmatory trial, the most effective dose is needed to be identified.

**Methods:**

The primary endpoint of a phase II trial is typically a binary endpoint of success or failure. The EMAX model, ubiquitous in pharmacology research, was fit for many compounds and described the data well, except for a single compound, which had nonmonotone dose–response (Thomas et al., Stat Biopharmaceutical Res. 6:302-317 2014). To mitigate the risk of nonmonotone dose response one of the alternative options is a Bayesian hierarchical EMAX model (Gajewski et al., Stat Med. 38:3123-3138 2019). The hierarchical EMAX adapts to its environment.

**Results:**

When the dose-response curve is monotonic it enjoys the efficiency of EMAX. When the dose-response curve is non-monotonic the additional random effect hyperprior makes the hierarchical EMAX model more adjustable and flexible. However, the normal dynamic linear model (NDLM) is a useful model to explore dose-response relationships in that the efficacy at the current dose depends on the efficacy of the previous dose(s). Previous research has compared the EMAX to the hierarchical EMAX (Gajewski et al., Stat Med. 38:3123-3138 2019) and the EMAX to the NDLM (Liu et al., BMC Med Res Method 17:149 2017), however, the hierarchical EMAX has not been directly compared to the NDLM.

**Conclusions:**

The focus of this paper is to compare these models and discuss the relative merit for each of their uses for an ongoing early phase dose selection study.

## Background

The primary objective of phase II design is to explore the dose-response curve (e.g. [1, 2]; and [3]) and find out the most effective dose for the subsequent phase III confirmative trial. The optimal dose, the dose level with the greatest probability of improvement in the rate of good outcome compared with the standard care is also determined. To identify the best dose, several statistical models have been proposed. Specifically, we are going to compare three statistical models using an illustrative example, the HOBIT trial [4]. Then, simulation is used to investigate operating characteristics of different designs of the HOBIT trial with the goal to select the treatment arm which is most likely to perform better than the control arm.

The EMAX model has been found to be a valid model across many compounds in pharmacology research [5]. The EMAX model is a monotonic model that has also found utility in pharmacodynamics [6]. However, there is at least one compound found in Thomas et al. [5] in which the monotonicity assumption did not hold. Therefore, to address the risk of nonmonotonicity Gajewski et al. [4] present the Bayesian hierarchical EMAX model. The hierarchical EMAX has an additional random effect hyperprior to support a more adjustable and flexible model. The hierarchical EMAX adapts to its environment. When the true dose-response relationship is truly monotonic it enjoys the efficiency of the monotonic EMAX. Additionally, however, when the true dose-response is non-monotonic it enjoys flexibility to follow the correct pattern. In the past, however, the normal dynamic linear model (NDLM) has been a useful model to explore a nonmonotonic dose-response relationship in that the efficacy at the current dose depends on the efficacy of the previous dose(s). While previous research has compared the EMAX to the hierarchical EMAX [4] and the EMAX to the NDLM [7], the hierarchical EMAX has not been directly compared to the NDLM. More specifically, in previous research it has been found that under monotonicity the EMAX is better than hierarchical EMAX and NDLM; but under nonmonotonicity the hierarchical EMAX and NDLM are better than EMAX. Specifically, the hierarchical EMAX enjoys a compromise and shared benefits of EMAX and independent models and is preferred under assumed monotonicity but a risk of nonmonotonicity. It is an excellent prespecified model for phase II designs. The focus of this paper is to explicitly compare the hierarchical EMAX to the NDLM and discuss their relative merits for use in an early phase dose selection study.

To be specific, the hierarchical EMAX model [4], simple NDLM, and 2nd order NDLM order are explored and compared. NDLM was originated in time series modeling and it is a method for model smoothing using the information borrowed from neighboring doses [7]. It combines variability from two sources, observational and system [8]. Furthermore, both 1st order and 2nd order NDLM are to be applied so that it can be seen which NDLM does better for selecting the most effective drug. In Section 2, we introduce the motivating trial and describe the models in detail along with Bayesian quantities. The application and evaluation of the models on simulated data are demonstrated in Section 3, with conclusions in Section 4.

## Method

### Motivating trial

The motivating study is the Hyperbaric Oxygen Brain Injury Treatment (HOBIT) trial. This is a phase II Bayesian clinical trial for selecting the best dose of hyperbaric oxygen treatment, which produces the greatest improvement in the rate of good neurological outcome versus standard of care for subjects with severe traumatic brain injury (TBI). A second goal of this phase II trial is to determine whether there is any hyperbaric treatment that has at least a 50% probability of demonstrating improvement in the rate of good neurological outcome versus a standard treatment in a subsequent phase III confirmatory trial, assuming 500 in the control and 500 in the arm treated with the selected optimal dose regimen of hyperbaric oxygen [4]. The allocation of this phase II trial has a fixed allocation of 20% subjects to control and equal allocation of the 80% to the seven active arms. When there is more than one active arm, typically, there are fewer patients on each of the active doses than on the control. This is done in order to optimize the power of the study. The total sample size is 200 subjects.

### Dose

Two factors of treatment are considered in the design of dose. To be specific, 4 levels of atmospheric pressure, 1.0,1.5 2.0, and 2.5 ATA were used. Another factor is whether 100% normobaric oxygen (NBH) is added or not. The dose was defined as a singular monotonic dose as a function of the total oxygen toxicity acquired during treatment. Table 1 summarizes the eight treatment arms involved in the trial. Dose strength as defined in Table 1 is the daily oxygen toxicity units per 100 (OTU/100) [4]. Table 1 below displays the conditions for each active treatment arm and dose strength.

**Table 1:** Conditions for each active treatment arm and dose strength. The control arm is modeled separately since standard of care dose not have a known OTU

Dose index *d*	Arm Name	OTUs ***ν***_***d***_***∗*** 100	Dose strength ***ν***_***d***_
*d* = 1	Control (1.0 ATA)	N/A*	N/A*
*d* = 2	1.5 ATA	260	*ν*_2_=2.60
*d* = 3	2 ATA	417	*ν*_3_=4.17
*d* = 4	NBH (100% FiO2 at 1.0 ATA)	540	*ν*_4_=5.40
*d* = 5	2.5 ATA	592	*ν*_5_=5.92
*d* = 6	1.5 ATA + NBH	620	*ν*_6_=6.20
*d* = 7	2 ATA + NBH	776	*ν*_7_=7.76
*d* = 8	2.5 ATA + NBH	952	*ν*_8_=9.52

### Models

This section introduces the three models considered: hierarchical EMAX model, simple NDLM, and 2nd order NDLM. For the Bayesian hierarchical EMAX model, a drift parameter is to be used to allow for more adjustment depending on the data. Furthermore, both 1st order and 2nd order NDLM are to be applied so that it can be seen which NDLM does better for selecting the most effective drug.

The probability an individual subject has a favorable outcome, *P*_*d*_, is modeled for each dose, where dose is indexed *d* ∈ {1, …, 8}. We use *ν*_*d*_ ∈ {N/A, 2.6, 4.17, 5.4, 5.92, 6.2, 7.76, 9.52} as the effective dose strength. The probability of a favorable outcome across doses is modeled with three different dose-response models. Assume all the subjects randomized to dose index *d* have a summed binomial outcome *Y*_*d*_:

$$ {Y}_d\sim Binomial\left({n}_d,{P}_d\right). $$

The log-odds of the probability of favorable outcomes, ${\theta}_d=\mathit{\log}\left(\frac{P_d}{1-{P}_d}\right)$, is modeled. In addition, for all models the single control arm (indexed *d* = 1) is modeled separately from the active doses and has a prior distribution of *θ*_1_~*N*(−.41, .75^2^). This vague prior on the *P*_1_ scale has a median of 0.40 and 95% equal-tailed interval of .09–.83 [4].

#### Hierarchical EMAX model

The hierarchical EMAX model is the following

$$ {\theta}_d={\phi}_1+\frac{\phi_2{\nu}_d}{\nu_d+{\phi}_3}+{\psi}_d,\kern3em d\in \left\{2,\dots, 8\right\}. $$

The hierarchical EMAX has EMAX parameters *ϕ*_*1*_, *ϕ*_*2*_, and *ϕ*_*3*_, as well as hierarchical parameters *ψ*_2_, *ψ*_3_,…, *ψ*_8_, and ${\phi}_4^2$:

- *ϕ*_*1*_ is a constant offset, and the logistic response when the effective dose strength is 0. The prior distribution is *ϕ*_1_ ∼ *N*(−0.41,1^2^).
- *ϕ*_*2*_ is a scalar coefficient of the fraction of the response due to the effective dose strength. It is the theoretical maximum effect above the constant offset that can be achieved. The prior distribution is *ϕ*_2_ ∼ *N*(0, 5^2^).
- *ϕ*_*3*_ is a positive scalar representing the effective dose strength that achieves 50% of the theoretical maximal effect. The prior distribution is *ϕ*_3_ ∼ *N*^+^(3, 10^2^). The notation *N*^+^ represents a positively truncated normal distribution.
- *ψ*_*d*_ is the off-curve effect that allows for a more flexible model (e.g. nonmonotone) and is modeled hierarchically ${\psi}_d\sim N\left(0,{\phi}_4^2\right)$, *d* ∈{ 2,…,8}. The variance parameter is modeled ${\phi}_4^2\sim Inverse- Gamma\left(\mathrm{0.1,0.001}\right)$ and its specification is critical.

The off-curve effect parameters are constrained such that ∑*ψ*_*d*_ = 0. The advantage of adding the random effect modeling is that when the EMAX provides a good fit to the data the random effect parameters, *ψ*_*d*_, are shrunk toward 0, on the other hand, when there are significant deviations from the EMAX model, the hyperparameter ${\phi}_4^2$ will be larger and therefore is less shrinkage towards the EMAX model, allowing the individual dose effects to create a custom fit [4].

#### Simple NDLM

It is a first order simple dynamic linear model since the current state depends on the previous one, except for the first active dose (*d* = 2):

$$ {\theta}_2\sim N\left(-.41,{.75}^2\right). $$

Then after that (*d* > 2):

$$ {\theta}_d\sim N\left({\theta}_{d-1},{\tau}_{d-1}^2\right), $$

where *θ*_*d* − 1_ represents the previous mean and ${\tau}_{d-1}^2$ represents the variance from the previous stage, specifically:

$$ {\tau}_d^2={\tau}^2\left({v}_{d+1}-{v}_d\right), $$

and

$$ {\tau}^2\sim IG\left(\frac{\tau_n}{2},\frac{\tau_u^2{\tau}_n}{2}\right) $$

*τ*_*u*_ is the prior central value and *τ*_*n*_ is the hierarchical prior weight. We let the prior central value to be *τ*_*u*_= 0.2 and prior weight to be *τ*_*n*_= 0.1, chosen to encourage smoothness from neighboring doses.

#### Second order NDLM

The next model to be considered is the second order (2nd) NDLM. It is second order because the current state depends on previous two states. To be specific, the parameter *θ*_*d*_ depends on the previous two stages, where involves *θ*_*d* − 1_, *θ*_*d* − 2_ and the dose strengths *v*_*d* − 1_ and *v*_*d* − 2_. The control is modeled separately as before and then the first active dose (*d* = 2):

$$ {\theta}_2\sim N\left(-.41,.{75}^2\right). $$

Then after that (*d* > 2):

$$ {\theta}_d=\left(\frac{\theta_{d-1}-{\theta}_{d-2}}{v_{d-1}-{v}_{d-2}}+{\zeta}_d\right)\left({v}_d-{v}_{d-1}\right)+{\theta}_{d-1}, $$

where

$$ {\zeta}_d\sim N\left(0,{\tau}_2^2\right), $$

and

$$ {\tau}_2^2\sim IG\left(\frac{\tau_n}{2},\frac{\tau_u^2{\tau}_n}{2}\right). $$

Where *τ*_*u*_ = .1 is the prior central is value, and *τ*_*n*_ = .2 is the hierarchical prior weight.

### Bayesian quantities of interest

We are interested in three Bayesian quantities, specifically, they are: the probability that each active dose is the maximal effective dose; the probability that each active dose performs better than the standard treatment (control group) and the predictive probability a dose would do better in a phase III trial compared to the standard treatment. These Bayesian quantities are used to draw conclusions.

#### Posterior distribution of treatment difference

This is the probability that the dose is superior to control, Pr(*P*_*d*_–*P*_*1*_ > 0) is calculated for each active dose using OpenBUGS (Appendix). The estimate of this quantity is the proportion of MCMC samples in which *P*_*d*_ > *P*_*1*_.

#### Maximum effective dose

This is the dose with the greatest probability of a better outcome. The posterior probability each dose is the maximum effective dose Pr(*D*_*Max*_) is calculated as the frequency of the MCMC samples in which each dose is the maximum.

#### Posterior predictive probability of future trial success

A future phase III trial is a fixed design with 500 subjects in control and 500 subjects in one of the best active dose selected from phase II. For each dose, the predictive probability of success in future trial is found by Pr(Phase III success; *n* = 500, α = 0.025), and the Type I error rate is one-sided α = 0.025. For each dose it is calculated by averaging power function over the posterior distribution for each dose. Therefore, the treatment effect and uncertainty is formally incorporated [4].

#### Final evaluation criteria

At the final analysis, the trial is considered successful if all of the following criteria are satisfied:

Pr(*P*_*d*_ > *P*_1_) > *β* for *d* = *greatest* Pr(*D*_*Max*_), and

Pr(Phase III Success; *n* = 500,*α* = 0.025) > 0.5 for *d* = greatest Pr(*D*_*Max*_).

Here *β* is the lower bound cutoff for trial success. Type I error rate changes depending on the choice of model for fixed *β* for the final analysis. In order to make sure that all models have the same type I error rate, which is set to be 10%, *β* will vary by the choice of model used. To provide 10% type I error rates across models, *β* is set to 0.922, 0.903, 0.938 for hierarchical EMAX, simple NDLM, 2nd order NDLM respectively, thus allowing for fair comparisons, these values were determined through simulation trial and error.

## Results

### Illustrative example

In this section, three models are used for the purpose of comparison, they are hierarchical EMAX model, simple NDLM, and second order NDLM. An illustrative example, Hyperbaric Oxygen Brain Injury Treatment trial, is used as motivation. It is a phase II clinical trial. The goal is to find out the optimal dose, which is defined as the dose regime with the greatest probability of improvement in the rate of good neurological outcome versus the standard care for patients with severe traumatic brain injury. The second goal is to find out the hyperbaric oxygen regime with at least 50% probability to demonstrate improvement in rate of good neurological outcome versus the control in the upcoming phase III confirmatory clinical trial given that 500 in the control and 500 in the selected optimal treatment arm [4].

The primary endpoint occurs 6 months after randomization called the sliding dichotomized severity adjusted GOS-E (favorable outcome is a 1 and unfavorable is a 0). Each patient will be randomized to control or one of seven active comparisons. The eight arms involve atmospheric pressures (1.0, 1.5, 2.0 and 2.5 atm absolute (ATA)) with or without additional 100% normobaric oxygen (NBH). Note that control is 1.0 ATA without NBH. In summary, there is a control group plus seven novel therapies each expressed by their respective dose of oxygen toxicity units (OTU) [4].

Three simulated datasets (scenarios) are used to compare the effect of each model. Namely, large monotone effect, NBH only effect, and over dose effect. For the large monotone effect, a monotonic increasing with the dose strength is assumed. For NBH effect, higher response rate takes place only in the treatment arms with additional 100% hyperbaric oxygen. Then for overdose effect, there is a monotonic increase of effect until the dose reaches the moderate level then the effect starts to go back down.

**Fig. 1:**
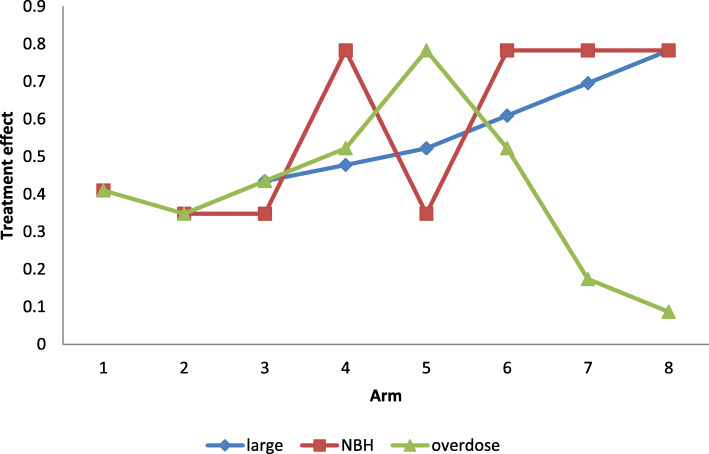
Three scenarios reflecting different dose-response relationships

The following plots illustrate the distribution of the three datasets, as it is shown below, the green line represents NBH effect, the normobaric oxygen only takes place in arms 4, 6, 7, and 8 because additional 100% normobaric oxygen is added. And the red line represents overdose effect. The green line is an upside-down “U” shaped curve since drug toxicity prevails with the increasing dose. Figure 1 is the graphical representation of the three scenarios for exploration of posterior distribution for assumed response. Table 2 is the summary of example simulated observed response under large monotone, NBH only, and overdose effects.

**Table 2:** Three different hypothetical datasets representing different dose-responses

	Dose Strength	d = 1 Control	d = 2 2.60	d = 3 4.17	d = 4 5.40	d = 5 5.92	d = 6 6.20	d = 7 7.76	d = 8 9.52
	n	39	23	23	23	23	23	23	23
**Large Monotone**									
**Response**	y	16	8	10	11	12	14	16	18
**%Response**	100*y/n	41.0%	34.8%	43.5%	47.8%	52.2%	60.9%	69.6%	78.3%
**NBH Only**									
**Response**	y	16	8	8	18	8	18	18	18
**%Response**	100*y/n	41.0%	34.8%	34.8%	78.3%	34.8%	78.3%	78.3%	78.3%
**Over-Dose**									
**Response**	y	16	8	10	12	18	12	4	2
**%Response**	100*y/n	41.0%	34.8%	43.5%	52.2%	78.3%	52.2%	17.4%	8.7%

#### Large monotone effect

**Fig. 2:**
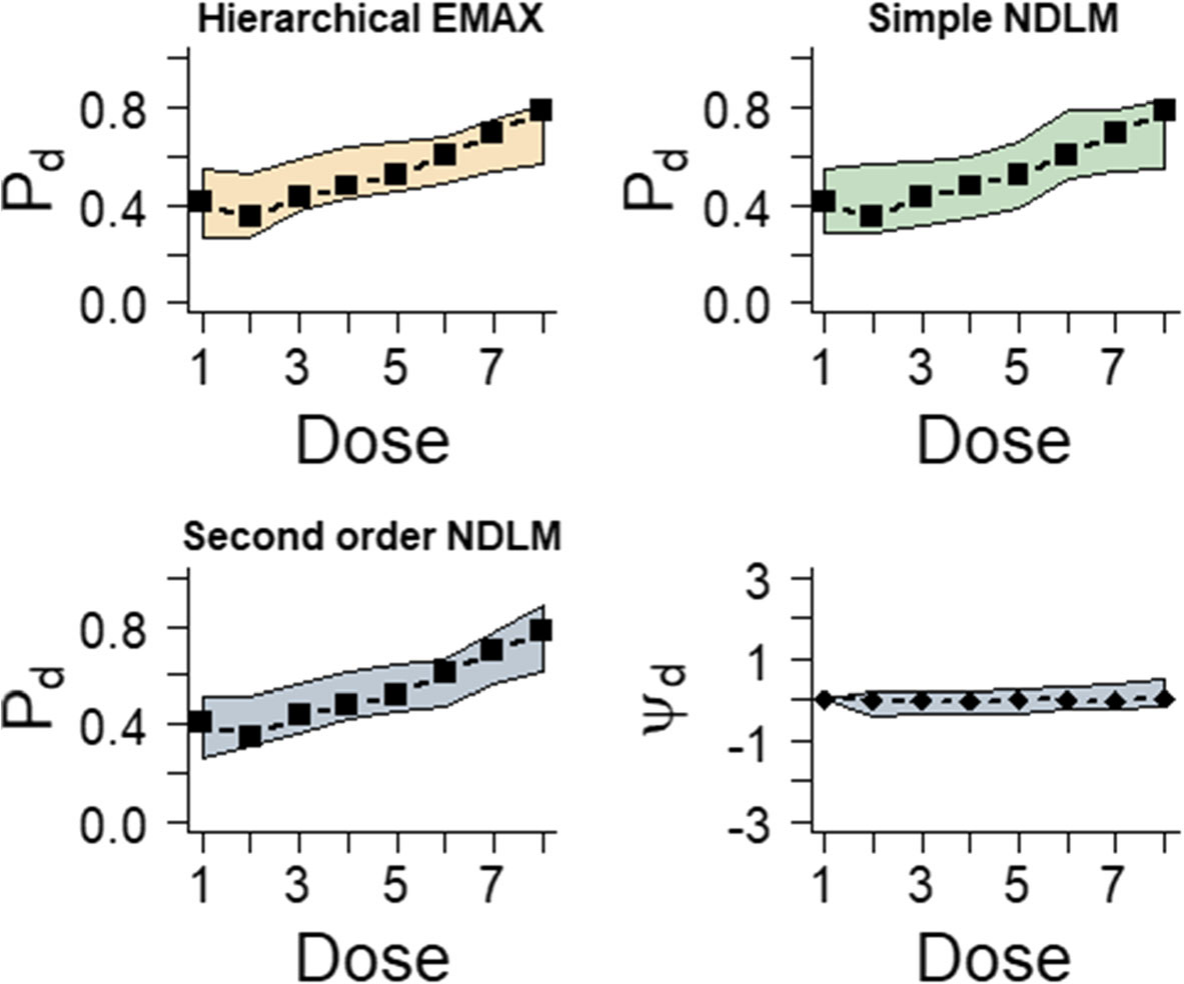
Results for fitting models in the large monotone effect example. The black squares in the first three frames represent the observed rate and the shaded regions are the 2.5% percentile and 97.5% percentile from models, which is the 95% credible interval for *P*_*d*_ for all three models. The last frame shows the 50%percentile point estimate and 2.5% percentile and 97.5% percentile for *ψ*_*d*_ in the hierarchical EMAX model

The section compares the results obtained using all three models and gives graphical representation of how they perform. As it is shown in Fig. 2, the simple NDLM has a wider credible interval than the hierarchical EMAX model and 2nd order NDLM. The monotonic increasing trend of response and the observed response rates are covered by all models. Table 3 is the summary of Bayesian quantities for model fitting in large monotone effect. All the models indicate that *d* = greatest Pr(Dmax) = 8, which has an effective dose strength 9.52, and the posterior probability that it performs better than the control is 1 for each of the models. And at this dose all the models have Bayesian quantities that lead to trial success since all Pr(Pd > Pl) > 0.922, 0.903, 0.938 for hierarchical EMAX mode, simple NDLM, 2nd order respectively. And they all have the future trial success probability greater than 0.5 for *d* = 8. Therefore, to sum up, hierarchical EMAX model and 2nd order model have better precision.

**Table 3:** Bayesian quantity results from fitting the large monotonic effect example

Large monotonic effect	***d*** = 1 Control	***d*** = 2 2.60	***d*** = 3 4.17	***d*** = 4 5.40	***d*** = 5 5.92	***d*** = 6 6.20	***d*** = 7 7.76	***d*** = 8 9.52
**pMAX**								
Hierarchical	0.00	0.00	0.00	0.00	0.00	0.01	0.08	0.90
simple NDLM	0.00	0.00	0.00	0.00	0.02	0.13	0.24	0.61
2nd order NDLM	0.00	0.00	0.00	0.00	0.00	0.00	0.03	0.97
**Pr(Pd > Pl)**								
Hierarchical	0.00	0.37	0.63	0.75	0.84	0.95	0.99	1.00
simple NDLM	0.00	0.59	0.68	0.78	0.90	0.99	0.00	1.00
2nd order NDLM	0.00	0.80	0.88	0.96	0.98	0.99	1.00	1.00
**Pr(phaseIII success)**								
Hierarchical	0.03	0.21	0.44	0.56	0.69	0.86	0.96	0.99
simple NDLM	0.02	0.33	0.43	0.54	0.72	0.98	0.97	0.98
2nd order NDLM	0.03	0.49	0.70	0.93	0.99	0.93	0.99	1.00

#### NBH only effect

**Fig. 3:**
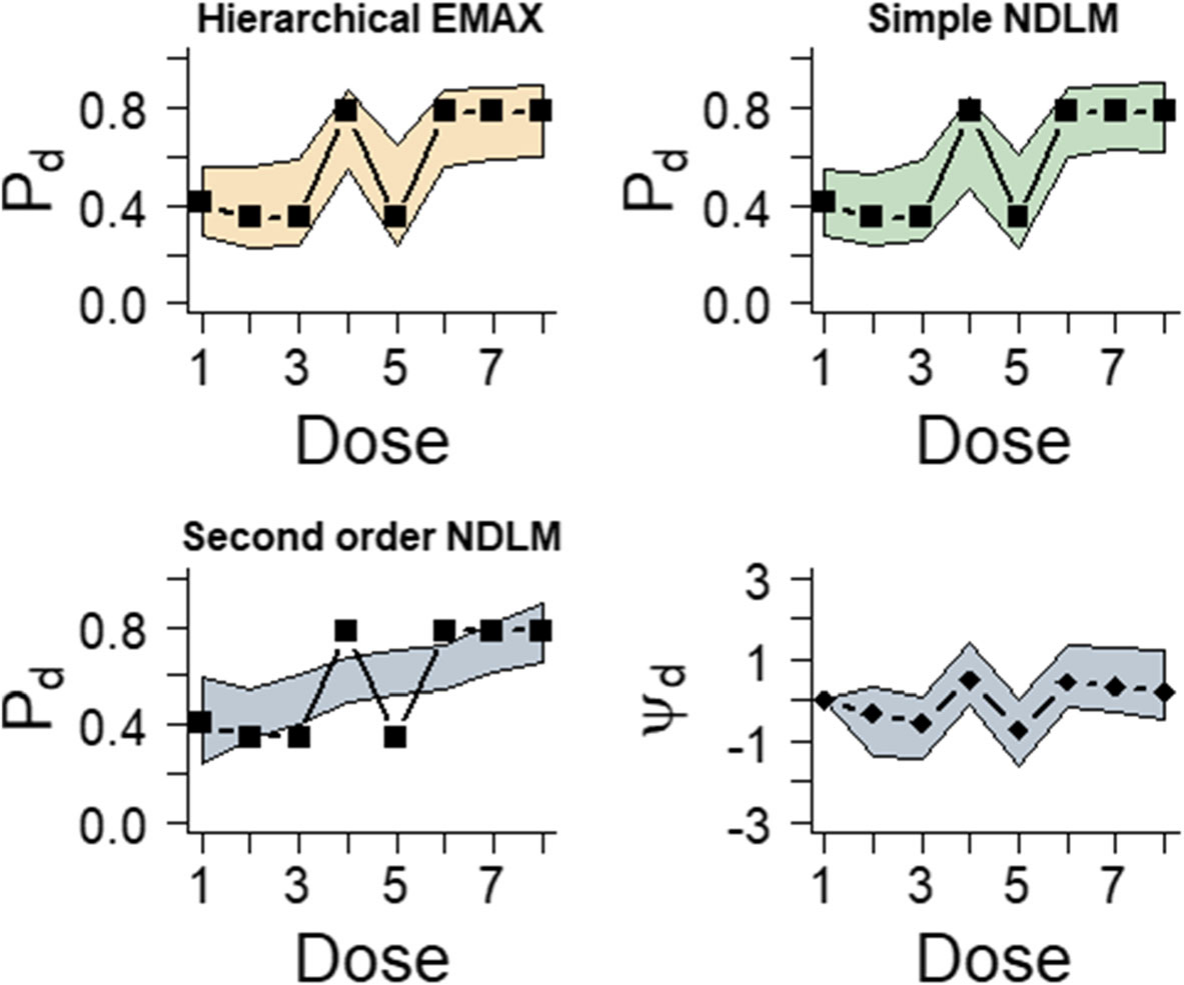
Results for fitting models in the NBH only effect example. The black squares in the first three frames represent the observed rate and the shaded regions are the 2.5% percentile and 97.5% percentile from models, which is the 95% confidence interval for *P*_*d*_ for all three models. The last frame shows the 50%percentile point estimate and 2.5% percentile and 97.5% percentile for *ψ*_*d*_ in the hierarchical EMAX model

This section is to compare the results obtained from hierarchical EMAX mode, simple NDLM, and second order NDLM under NBH condition. Figure 3 illustrates the observed response rate and 95% credible interval for four models. In this scenario, the hierarchical EMAX model and simple NDLM cover all the observed rates but they both have wider credible intervals compared to 2nd order NDLM. However, 2nd order NDLM does not represent non-linear effect. To be specific, 2nd order NDLM underestimates treatment 4 and 6 since the observed response rates are above the 95% credible interval. And it overestimates treatment 3 and 5 in that the credible intervals are well above the observed response rate. By contrast, hierarchical EMAX model and simple NDLM do well in that they both cover observed response rate though they have wider credible intervals compared to 2nd order NDLM. The reason is that the adding off-curve effect is larger than zero at each four NBH doses, which is as displayed by the plot. Table 4 displays the results of Bayesian quantities of model fitting in the NBH only effect.

**Table 4:** Bayesian quantity results from fitting the NBH effect example

Large monotonic effect	***d*** = 1 Control	***d*** = 2 2.60	***d*** = 3 4.17	***d*** = 4 5.40	***d*** = 5 5.92	***d*** = 6 6.20	***d*** = 7 7.76	***d*** = 8 9.52
**pMAX**								
Hierarchical	0.00	0.00	0.00	0.16	0.00	0.18	0.25	0.41
simple NDLM	0.00	0.00	0.00	0.05	0.00	0.26	0.30	0.39
2nd order NDLM	0.00	0.00	0.00	0.00	0.00	0.00	0.04	0.96
**Pr(Pd > Pl)**								
Hierarchical	0.00	0.38	0.40	1.00	0.42	1.00	1.00	1.00
simple NDLM	0.00	0.41	0.55	0.98	0.57	1.00	1.00	1.00
2nd order NDLM	0.00	0.85	0.94	1.00	1.00	1.00	1.00	1.00
**Pr(phaseIII success)**								
Hierarchical	0.03	0.22	0.24	1.00	0.25	0.99	0.99	1.00
simple NDLM	0.03	0.21	0.34	0.93	0.38	1.00	1.00	1.00
2nd order NDLM	0.03	0.61	0.80	0.95	0.98	1.00	1.00	1.00

#### Overdose effect

**Fig. 4:**
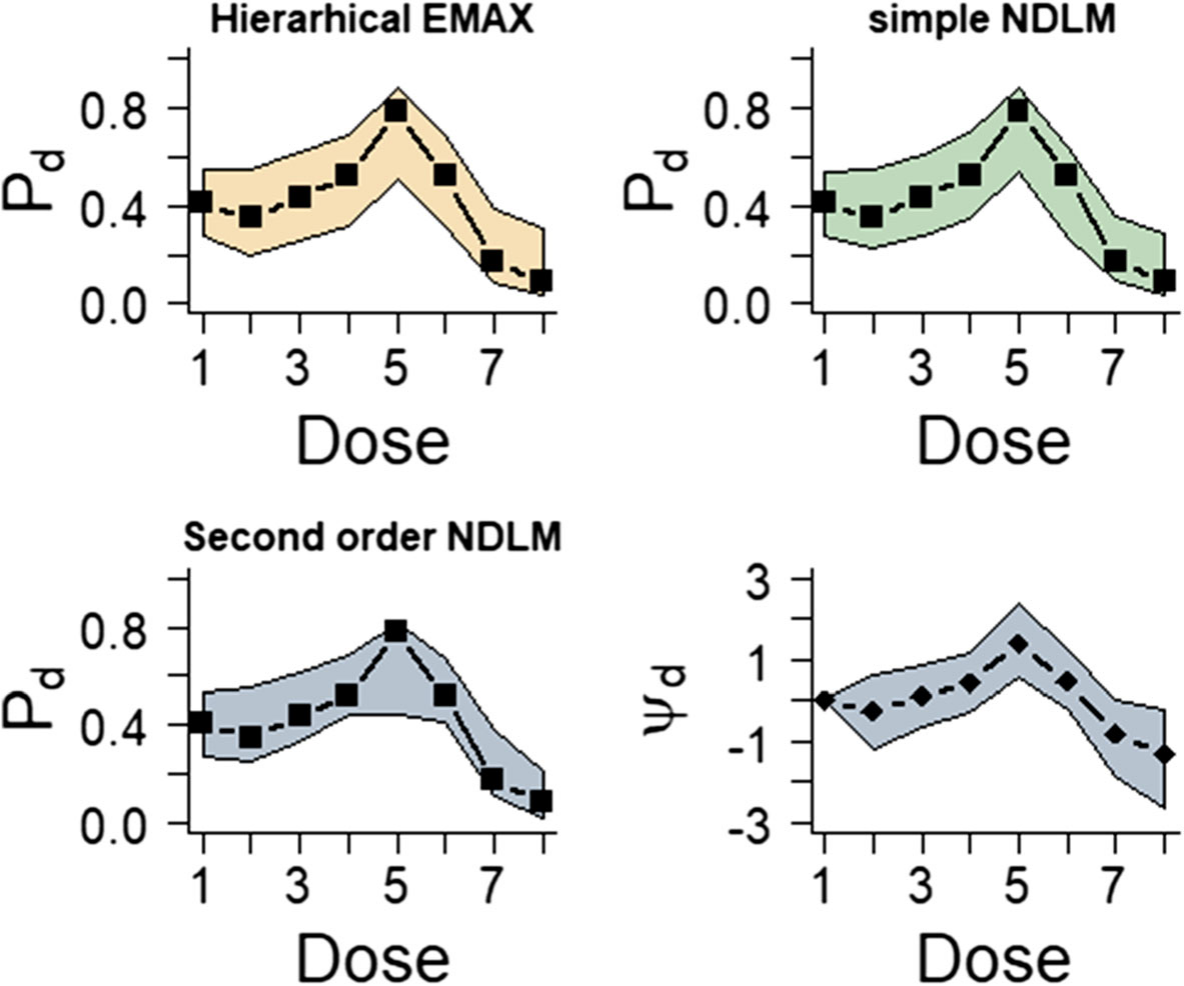
Results for fitting models in the overdose effect example. The black squares in the first three frames represent the observed rate and the shaded regions are the 2.5% percentile and 97.5% percentile from models, which is the 95% confidence interval for *P*_*d*_ for all three models. The last frame shows the 50%percentile point estimate and 2.5% percentile and 97.5% percentile for *ψ*_*d*_ in the hierarchical EMAX model

Figure 4 displays the 95% credible intervals with the observed response rate for the overdose example. Both hierarchical EMAX model and simple NDLM cover the entire observed rate. On the contrary, 2nd order NDLM fails to respond to the nonlinear effect in the middle in that it underestimates the effect of treatment 5. The reason why hierarchical EMAX well represent the nonlinear response is that the off-curve random term bumps up at the maximum effective dose strength at *d* = 5 with *v*_*d*_ =5.92, but the 2nd order NDLM covered the observed response rate at *d* = 5. Table 5 displays the results of Bayesian quantities of model fitting in the NBH only effect.

**Table 5:** Bayesian quantity results from fitting the overdose effect example

Large monotonic effect	***d*** = 1 Control	***d*** = 2 2.60	***d*** = 3 4.17	***d*** = 4 5.40	***d*** = 5 5.92	***d*** = 6 6.20	***d*** = 7 7.76	***d*** = 8 9.52
**pMAX**								
Hierarchical	0.00	0.00	0.01	0.03	0.92	0.04	0.00	0.00
simple NDLM	0.00	0.00	0.01	0.04	0.95	0.01	0.00	0.00
2nd order NDLM	0.00	0.03	0.11	0.33	0.46	0.07	0.00	0.00
**Pr(Pd > Pl)**								
Hierarchical	0.00	0.32	0.56	0.79	1.00	0.78	0.03	0.00
simple NDLM	0.00	0.41	0.62	0.86	1.00	0.64	0.03	0.00
2nd order NDLM	0.00	0.61	0.77	0.96	0.96	0.92	0.07	0.00
**Pr(phaseIII success)**								
Hierarchical	0.03	0.18	0.37	0.61	0.98	0.60	0.01	0.00
simple NDLM	0.25	0.21	0.41	0.69	0.99	0.44	0.01	0.00
2nd order NDLM	0.03	0.40	0.59	0.86	0.87	0.79	0.02	0.00

### Simulation study

This purpose of this section is to use simulations to obtain operating characteristics of trial designs, such as the probability of selecting a correct arm (an arm that is correctly better than control) and the probability of selecting the best arm. To be specific, Fixed and Adaptive Clinical Trial Simulator 6.2 (FACTS) (Berry Consultants, Austin, TX) is used to study the characteristics of the three models (Hierarchical EMAX, simple NDLM, and second order NDLM). The shaded region in Table 6 represents the treatment arms expected to perform better than the control, as well as the absolute best in **bold**.

**Table 6:** The arms expected to perform better than the control, represented by the shaded region. The best arm(s) is in **underlined**

Effect	***d*** = 1 Control	***d*** = 2 2.60	***d*** = 3 4.17	***d*** = 4 5.40	***d*** = 5 5.92	***d*** = 6 6.20	***d*** = 7 7.76	***d*** = 8 9.52
Large	0.40	0.59	0.60	0.61	0.62	0.63	0.64	**0.65**
NBH	0.40	0.40	0.40	**0.70**	0.40	**0.70**	**0.70**	**0.70**
Over Dose	0.40	0.40	0.50	0.55	**0.70**	0.40	0.35	0.30

### Probability of selecting a correct arm

The probability of selecting a correct arm is the probability of selecting the treatment arms which are expected to perform better than the control group. Specifically, for large monotone effect, all the 7 treatment arms are expected to have a higher response rate than that of control, therefore the probability of selecting a correct groups is the probability that any of the 7 treatment arms are chosen. For NBH effect, since 100% additional normbaric oxygen is added to group 4, 6, 7 and 8 treatment, then theses arms are expected to have a higher response rate. Then for overdose effect, toxicity is taken into consideration, drug toxicity prevails with the increasing dose. Therefore arm 3, 4, 5 are expected to have a better performance.

**Fig. 5:**
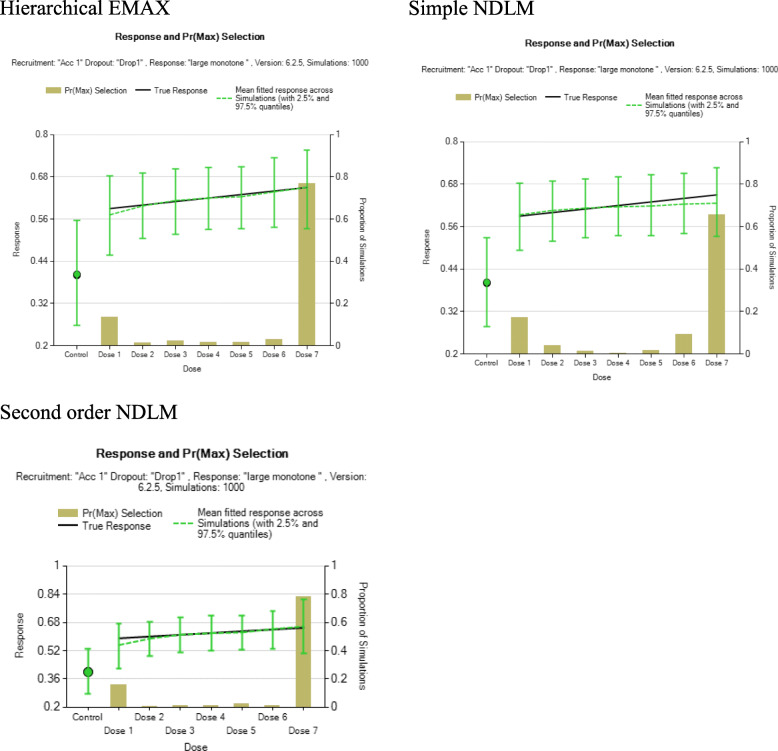
Summary of results from 1000 simulated trials using the dose-response relationship scenario under the assumption of “Large monotone effect” and analyzed using three different models: hierarchical EMAX; Simple NDLM; and Second order NDLM. Shown is the Pr(*D*_*Max*_), the true response, and the posterior mean of fitted response, each as a function of control arm and dose

Figure 5 shows the results of Pr(*D*_*Max*_) selection among all the models, for large monotone effect. They all have a high probability of selecting Dose 7 to be the best among active doses with hierarchical EMAX and 2nd order NDLM doing better in selecting the Dose 7 than simple NDLM because they both use more information from the other doses.

**Fig. 6:**
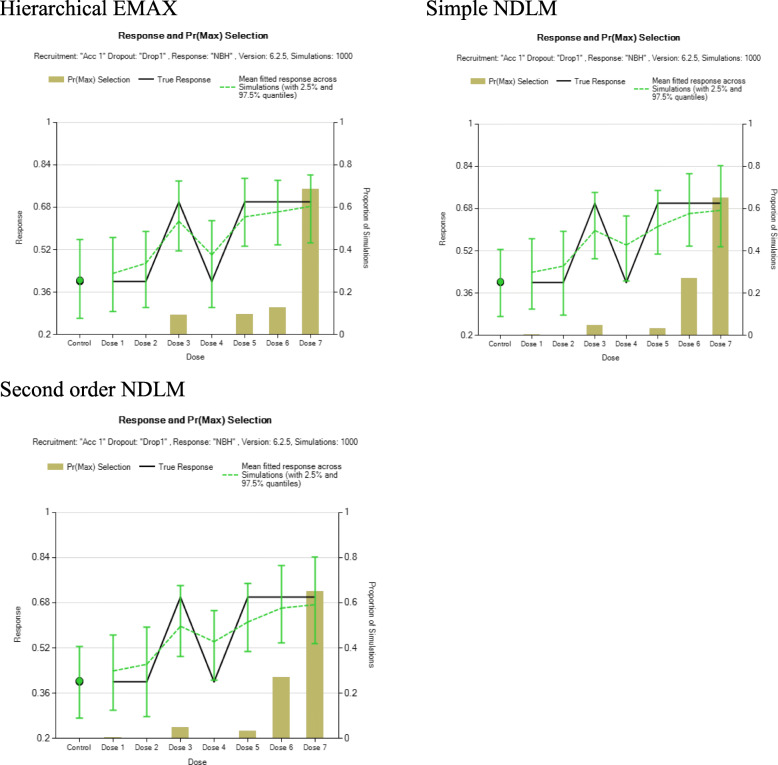
Summary of results from 1000 simulated trials using the dose-response relationship scenario under the assumption of “NBH only effect” and analyzed using three different models: hierarchical EMAX; Simple NDLM; and Second order NDLM. Shown is the Pr(*D*_*Max*_), the true response, and the posterior mean of fitted response, each as a function of control arm and dose

In the NBH only effects, in Fig. 6, the probability for each model of selecting a correct arm (Doses 3, 5, 6, or 7) are roughly the same, all of them have leans towards Dose 7.

**Fig. 7:**
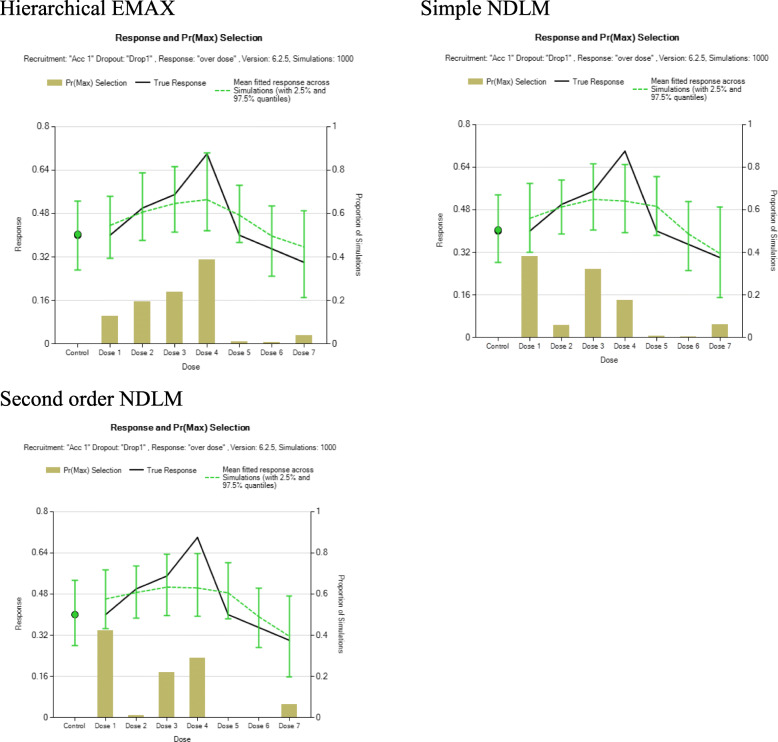
Summary of results from 1000 simulated trials using the dose-response relationship scenario under the assumption of “Overdose effect” and analyzed using three different models: hierarchical EMAX; Simple NDLM; and Second order NDLM. Shown is the Pr(*D*_*Max*_), the true response, and the posterior mean of fitted response, each as a function of control arm and dose

However, there is a noticeable divergence when it comes to overdose effect (Fig. 7). The hierarchical EMAX model has a greater probability of choosing the correct optimal dose (Dose 4) than both the simple NDLM and 2nd order NDLM. Further, the simple NDLM and the 2nd order NDLM both have higher probabilities of choosing a suboptimal dose (Dose 1) than does the hierarchical EMAX model. This is in fact consistent with the result obtained previously, which indicates that the simple NDLM and the 2nd order NDLM do not well represent the nonlinear effect. Figure 7 shows the hierarchical EMAX model well responds the nonlinear effect.

Table 7 below displays the probability of selecting a correct arm for each model. Specifically, for large monotone effect, we expect all the treatment arms to perform better than the control dose, therefore, the probability of selecting a correct arm is the probability that any of the treatment arms are chosen. The hierarchical EMAX model and simple NDLM both have the higher probability of selecting arms compared to 2nd order NDLM. For NBH only effect, we assume that four treatment arms, 4th, 6th, 7th and 8th, to be chosen since the additional 100% oxygen is added to these arms. Therefore, the probability of choosing a correct arm is the probability that any of the four arms are selected. Based on the result, it appears that all three models performed approximately equally well in that all the models have a probability well above 90%. However, the results diverge when it comes to overdose effect: as it is shown in Table 7, we can see that the probability of selecting a correct arm of hierarchical EMAX model is the highest among all the models (although simple NDLM is close). Further, 2nd order NDLM is less attractive because its probability of selecting an incorrect dose is much higher. This result is in fact consistent with the fact that nonlinear response is not well represented by either 2nd model NDLM.

**Table 7:** The probability for selecting a correct effective dose (n = 200). All designs are calibrated to have a Type I error rate of 10%

	Hier. EMAX		Simple NDLM		2nd order NDLM	
Effect	P(correct)	P(incorrect)	P(correct)	P(incorrect)	P(correct)	P(incorrect)
Large	**0.946**	0.000	**0.946**	0.000	0.878	0.000
NBH	0.949	0.001	**0.961**	0.001	0.941	0.000
overdose	**0.477**	0.067	0.442	0.062	0.296	0.105

### The probability for selecting the single best effective dose

This section is devoted to comparing the probability of selecting the maximum effective dose (e.g. the best among all correct doses). According to the result displayed in Table 8, we can see that the hierarchical EMAX model works the best among those three models since it either is has the greatest probabilities of detecting the best arm for each scenario compared to the rest or close. Consistently with the conclusion previously obtained, since the 2nd order NDLM did not well represent the nonlinear effect, the probabilities of selecting the best arm is much lower compared to hierarchical EMAX model and simple NDLM. This can be seen especially when it comes to overdose effect: the probability of selecting the maximum effective dose for legacy 2nd order NDLM, it is much lower than that of hierarchical EMAX model.

**Table 8:** The probability for selecting the maximum effective dose(n = 200). All designs are calibrated to have a Type I error rate of 10%

	Hier. EMAX		Simple NDLM		2nd order NDLM	
Effect	P(correct)	P(incorrect)	P(correct)	P(incorrect)	P(correct)	P(incorrect)
Large	**0.734**	0.212	0.674	0.272	0.697	0.181
NBH	0.949	0.001	**0.961**	0.001	0.941	0.000
overdose	**0.401**	0.143	0.232	0.272	0.100	0.289

### Ideal design percentage comparing models and literature

**Fig. 8:**
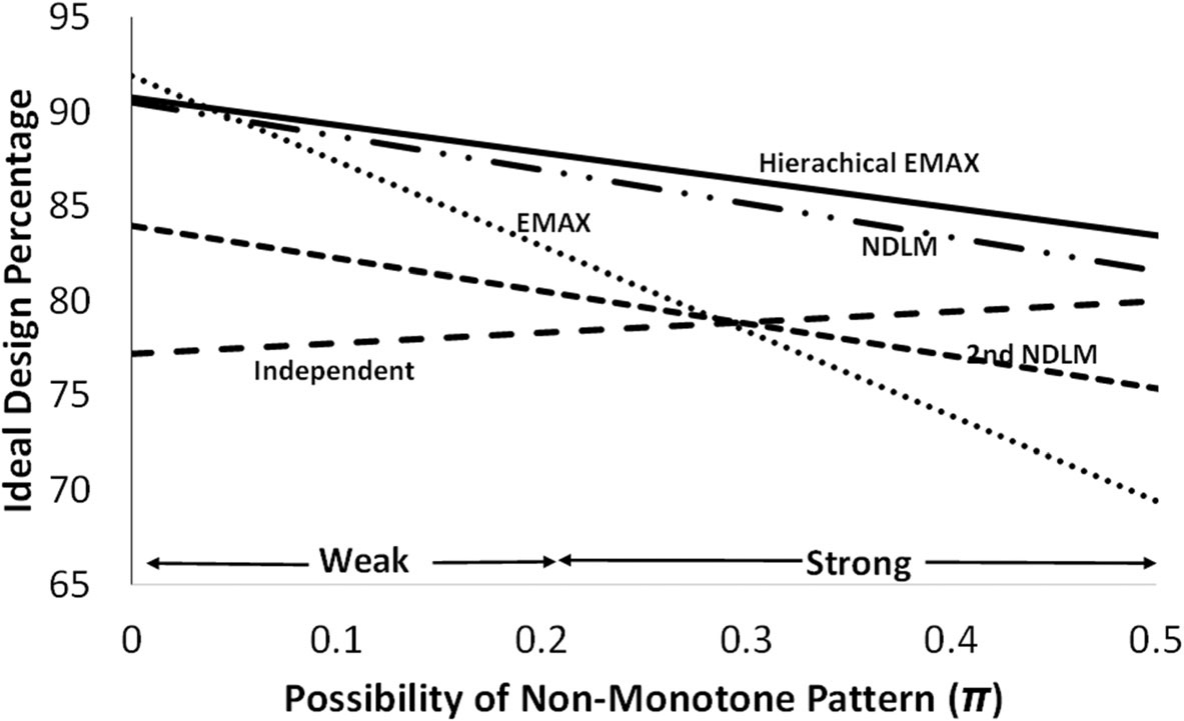
Comparison of models in this paper (hierarchical EMAX and the NDLMs) to models in the literature (independent and EMAX)

Presented is the ideal design percentage [9], the ratio of the *difference in the expected and the minimum true rat*e and the *difference in the maximum true rate and minimum true rate*, assuming that when a treatment is not successful the control arm is used in practice. The possibility of non-monotone pattern produces a combination of the effects Large, NBH Only, and Over Dose. Let *π* be the probability of a non-monotone pattern (this probability is split between the two non-monotone patterns NBH Only and Over Dose), ideal design percentage (ID), for each model is calculated as a function of the probability of the effects, therefore this operating characteristic becomes (1 − *π*)*ID*_*Large*_ + (*π*/2)*ID*_*NBH*_ + (*π*/2)*ID*_*Over Dose*_. The ID was calculated for all of the models in this paper as well as for EMAX and independent models [4]. The independent model has separate priors for each dose and does not assume any pattern, specifically is *θ*_*d*_ ∼ *N*(−0.41,1^2^), *d* ∈ {2, …, 8}. As in the previous work and shown in Fig. 8, no model is best across all possibilities of non-monotone patterns however hierarchical EMAX and NDLM models work very well across a broad range, with hierarchical EMAX having an edge over NDLM.

## Discussion

It has been found that both Bayesian hierarchical EMAX model and simple NDLM work well when the response curve is non-monotone. And they work equally well in terms of the probability of selecting the right dose. However, second order NDLM failed to react to the nonlinear spikes. Therefore, when the response it assumed to be nonmonotone, the higher order NDLM may not be a good option. Further, when it comes to the probability of selecting the right dose, hierarchical EMAX model and simple NDLM have a relatively higher probability compared with second order NDLM. And for the probability of selecting the best dose hierarchical EMAX model does the best compared to both simple NDLM and 2nd order NDLM. As for the reason why 2nd order NDLM failed to respond to the nonlinear spikes, it is because the current state is associated with the previous two states so the current status is more correlated with each other, and this makes a higher NDLM inaccurate when the response curve fluctuates.

## Conclusions

In conclusion, we have found in the HOBIT trial that hierarchical EMAX works better than the NDLM choices because it has better overall operating characteristics across monotone and nonmonotone cases.

## Supplementary information

**Additional file 1.** Appendix: WinBUGS code.

## Data Availability

Not applicable.

## Abbreviations

HOBIT: Hyperbaric oxygen brain injury treatment
EMAX: Efficacy maximum
NDLM: Normal dynamic linear model
TBI: Traumatic brain injury
ATA: Atmospheres absolute
NBH: Normobaric oxygen
OUT: Oxygen toxicity units
MCMC: Markov Chain Monte Carlo
NIH: National Institutes of Health
