# Supplementary material for: Comparison of hierarchical EMAX and NDLM models in dose-response for early phase clinical trials
Source: BMC Med Res Methodol. 2020 Jul 20;20:194. doi: 10.1186/s12874-020-01071-2 (PMC7370408; doi:10.1186/s12874-020-01071-2)
Supplement: Supplementary file 1 — Additional file 1. Appendix: WinBUGS code. [file 12874_2020_1071_MOESM1_ESM.docx]

**Appendix: WinBUGS code**

###Control Separately

####hierarchical EMAX

model

{

####Difference from Control

y[1]~dbin(P[1],n[1])

logit(P[1])<-theta[1]

theta[1]~dnorm(-.41,invV)

invV<-1/pow(.75,2)

####Difference from Control

thetadiff[1]<- -1000000

Pdiff[1]<- -1000000

for (d in 2:8)

{

###Active Dose Model

y[d]~dbin(P[d],n[d])

logit(P[d])<-theta[d]

theta[d]<-a[1]+a[2]*nu[d]/(nu[d]+a[3])+psi[d] ## replace “+psi[d]” with “#+psi[d]” to make Emax

##Repace with theta[d]~dnorm(-.41,1) for independent

####Difference from Control

thetadiff[d]<-theta[d]-theta[1]

Pdiff[d]<-P[d]-P[1]

}

a[1]~dnorm(-.41,1)

a[2]~dnorm(0,.04)

a[3]~dnorm(3,.01)I(0,) ####This is right

psi[1]<-0 ### Probably not necessary

psi_adj[1]<-0

for (d in 2:8)

{

psi_adj[d]~dnorm(0,inva24_adj)

psi[d]<-psi_adj[d]-mean(psi_adj[2:8])

}

inva24_adj<-6/7*inva24

inva24~dgamma(.1,.001)

a[4]<-sqrt(1/inva24)

####Probability max relative to control

diffMAX[1]<-thetadiff[1]-max(max(max(max(max(max(thetadiff[2],thetadiff[3]),thetadiff[4]),thetadiff[5]),thetadiff[6]),thetadiff[7]),thetadiff[8])

diffMAX[2]<-thetadiff[2]-max(max(max(max(max(max(thetadiff[1],thetadiff[3]),thetadiff[4]),thetadiff[5]),thetadiff[6]),thetadiff[7]),thetadiff[8])

diffMAX[3]<-thetadiff[3]-max(max(max(max(max(max(thetadiff[1],thetadiff[2]),thetadiff[4]),thetadiff[5]),thetadiff[6]),thetadiff[7]),thetadiff[8])

diffMAX[4]<-thetadiff[4]-max(max(max(max(max(max(thetadiff[1],thetadiff[2]),thetadiff[3]),thetadiff[5]),thetadiff[6]),thetadiff[7]),thetadiff[8])

diffMAX[5]<-thetadiff[5]-max(max(max(max(max(max(thetadiff[1],thetadiff[2]),thetadiff[3]),thetadiff[4]),thetadiff[6]),thetadiff[7]),thetadiff[8])

diffMAX[6]<-thetadiff[6]-max(max(max(max(max(max(thetadiff[1],thetadiff[2]),thetadiff[3]),thetadiff[4]),thetadiff[5]),thetadiff[7]),thetadiff[8])

diffMAX[7]<-thetadiff[7]-max(max(max(max(max(max(thetadiff[1],thetadiff[2]),thetadiff[3]),thetadiff[4]),thetadiff[5]),thetadiff[6]),thetadiff[8])

diffMAX[8]<-thetadiff[8]-max(max(max(max(max(max(thetadiff[1],thetadiff[2]),thetadiff[3]),thetadiff[4]),thetadiff[5]),thetadiff[6]),thetadiff[7])

for (d in 1:8)

{

pMAX[d]<-step(diffMAX[d])

pPBO[d]<-step(thetadiff[d])

pPBOf[d]<-step(Pdiff[d]-.1)

}

####Allocation Weights Done in Excel

#####Now do phase IIII success prediction

ntx<-500

nc<-500

yc~dbin(P[1],nc)

Phatc<-yc/nc

for (d in 1:8)

{

ytx[d]~dbin(P[d],ntx)

Phattx[d]<-ytx[d]/ntx

V[d]<-Phatc*(1-Phatc)/nc+Phattx[d]*(1-Phattx[d])/ntx

Z[d]<-(Phatc-Phattx[d])/sqrt(V[d])

pvalue[d]<-phi(Z[d])

PphaseIIIS[d]<-1-step(pvalue[d]-.025)

}

}

list(inva24=1)

#######For paper (n=200); 20% control, equal elsewhere

###Large effect

list(

n=c(39, 23, 23, 23, 23, 23, 23, 23),y=c( 16, 8, 10, 11,12,14,16,18),

nu=c(0, 2.6, 4.17, 5.4, 5.92, 6.2, 7.76, 9.52))

###NBH only:

list(n=c(39, 23, 23, 23, 23, 23, 23, 23),y=c(16,8,8,18,8,18,18,18),

nu=c(0, 2.6, 4.17, 5.4, 5.92, 6.2, 7.76, 9.52))

###Over dose

list(

n=c(39, 23, 23, 23, 23, 23, 23, 23),y=c( 16, 8, 10, 12, 18,12,4,2),

nu=c(0, 2.6, 4.17, 5.4, 5.92, 6.2, 7.76, 9.52))

model

{

###simple NDLM

####Difference from Control

y[1]~dbin(P[1],n[1])

logit(P[1])<-theta[1]

theta[1]~dnorm(-.41,invV)

invV<-1/pow(.75,2)

tau[1]<-invV

####Difference from Control

thetadiff[1]<- -1000000

Pdiff[1]<- -1000000

for (d in 2:8)

{

###Active Dose Model

y[d]~dbin(P[d],n[d])

logit(P[d])<-theta[d]

theta[d]~dnorm(theta[d-1],tau[d-1])

####Difference from Control

thetadiff[d]<-theta[d]-theta[1]

Pdiff[d]<-P[d]-P[1]

}

for (d in 2:7)

{ tau[d]<-(nu[d+1]-nu[d])*tao

}

tao~dgamma(.1,.001)

####Probability max relative to control

diffMAX[1]<-thetadiff[1]-max(max(max(max(max(max(thetadiff[2],thetadiff[3]),thetadiff[4]),thetadiff[5]),thetadiff[6]),thetadiff[7]),thetadiff[8])

diffMAX[2]<-thetadiff[2]-max(max(max(max(max(max(thetadiff[1],thetadiff[3]),thetadiff[4]),thetadiff[5]),thetadiff[6]),thetadiff[7]),thetadiff[8])

diffMAX[3]<-thetadiff[3]-max(max(max(max(max(max(thetadiff[1],thetadiff[2]),thetadiff[4]),thetadiff[5]),thetadiff[6]),thetadiff[7]),thetadiff[8])

diffMAX[4]<-thetadiff[4]-max(max(max(max(max(max(thetadiff[1],thetadiff[2]),thetadiff[3]),thetadiff[5]),thetadiff[6]),thetadiff[7]),thetadiff[8])

diffMAX[5]<-thetadiff[5]-max(max(max(max(max(max(thetadiff[1],thetadiff[2]),thetadiff[3]),thetadiff[4]),thetadiff[6]),thetadiff[7]),thetadiff[8])

diffMAX[6]<-thetadiff[6]-max(max(max(max(max(max(thetadiff[1],thetadiff[2]),thetadiff[3]),thetadiff[4]),thetadiff[5]),thetadiff[7]),thetadiff[8])

diffMAX[7]<-thetadiff[7]-max(max(max(max(max(max(thetadiff[1],thetadiff[2]),thetadiff[3]),thetadiff[4]),thetadiff[5]),thetadiff[6]),thetadiff[8])

diffMAX[8]<-thetadiff[8]-max(max(max(max(max(max(thetadiff[1],thetadiff[2]),thetadiff[3]),thetadiff[4]),thetadiff[5]),thetadiff[6]),thetadiff[7])

for (d in 1:8)

{

pMAX[d]<-step(diffMAX[d])

pPBO[d]<-step(thetadiff[d])

pPBOf[d]<-step(Pdiff[d]-.1)

}

####Allocation Weights Done in Excel

#####Now do phase IIII success prediction

ntx<-500

nc<-500

yc~dbin(P[1],nc)

Phatc<-yc/nc

for (d in 1:8)

{

ytx[d]~dbin(P[d],ntx)

Phattx[d]<-ytx[d]/ntx

V[d]<-Phatc*(1-Phatc)/nc+Phattx[d]*(1-Phattx[d])/ntx

Z[d]<-(Phatc-Phattx[d])/sqrt(V[d])

pvalue[d]<-phi(Z[d])

PphaseIIIS[d]<-1-step(pvalue[d]-.025)

}

}

model

{

###second order NDLM

####Difference from Control

y[1]~dbin(P[1],n[1])

logit(P[1])<-theta[1]

theta[1]~dnorm(-.41,invV)

invV<-1/pow(.75,2)

tau[1]<-invV

y[2]~dbin(P[2],n[2])

logit(P[2])<-theta[2]

theta[2]~dnorm(0, invV2)

invV2<-1/pow(.75,2)

tau[2]<-invV2

####Difference from Control

thetadiff[1]<- -1000000

Pdiff[1]<- -1000000

for (d in 3:8)

{

###Active Dose Model

y[d]~dbin(P[d],n[d])

logit(P[d])<-theta[d]

theta[d]<-(((theta[d-1]-theta[d-2])/(nu[d-1]-nu[d-2]))+zeta[d])*(nu[d]-nu[d-1])+theta[d-1]

zeta[d]~dnorm(0,tau2)

####Difference from Control

thetadiff[d]<-theta[d]-theta[1]

Pdiff[d]<-P[d]-P[1]

}

thetadiff[2]<-theta[2]-theta[1]

Pdiff[2]<-P[2]-P[1]

tau2~dgamma(.1,.001)

####Probability max relative to control

diffMAX[1]<-thetadiff[1]-max(max(max(max(max(max(thetadiff[2],thetadiff[3]),thetadiff[4]),thetadiff[5]),thetadiff[6]),thetadiff[7]),thetadiff[8])

diffMAX[2]<-thetadiff[2]-max(max(max(max(max(max(thetadiff[1],thetadiff[3]),thetadiff[4]),thetadiff[5]),thetadiff[6]),thetadiff[7]),thetadiff[8])

diffMAX[3]<-thetadiff[3]-max(max(max(max(max(max(thetadiff[1],thetadiff[2]),thetadiff[4]),thetadiff[5]),thetadiff[6]),thetadiff[7]),thetadiff[8])

diffMAX[4]<-thetadiff[4]-max(max(max(max(max(max(thetadiff[1],thetadiff[2]),thetadiff[3]),thetadiff[5]),thetadiff[6]),thetadiff[7]),thetadiff[8])

diffMAX[5]<-thetadiff[5]-max(max(max(max(max(max(thetadiff[1],thetadiff[2]),thetadiff[3]),thetadiff[4]),thetadiff[6]),thetadiff[7]),thetadiff[8])

diffMAX[6]<-thetadiff[6]-max(max(max(max(max(max(thetadiff[1],thetadiff[2]),thetadiff[3]),thetadiff[4]),thetadiff[5]),thetadiff[7]),thetadiff[8])

diffMAX[7]<-thetadiff[7]-max(max(max(max(max(max(thetadiff[1],thetadiff[2]),thetadiff[3]),thetadiff[4]),thetadiff[5]),thetadiff[6]),thetadiff[8])

diffMAX[8]<-thetadiff[8]-max(max(max(max(max(max(thetadiff[1],thetadiff[2]),thetadiff[3]),thetadiff[4]),thetadiff[5]),thetadiff[6]),thetadiff[7])

for (d in 1:8)

{

pMAX[d]<-step(diffMAX[d])

pPBO[d]<-step(thetadiff[d])

pPBOf[d]<-step(Pdiff[d]-.1)

}

####Allocation Weights Done in Excel

#####Now do phase IIII success prediction

ntx<-500

nc<-500

yc~dbin(P[1],nc)

Phatc<-yc/nc

for (d in 1:8)

{

ytx[d]~dbin(P[d],ntx)

Phattx[d]<-ytx[d]/ntx

V[d]<-Phatc*(1-Phatc)/nc+Phattx[d]*(1-Phattx[d])/ntx

Z[d]<-(Phatc-Phattx[d])/sqrt(V[d])

pvalue[d]<-phi(Z[d])

PphaseIIIS[d]<-1-step(pvalue[d]-.025)

}

}
